# Comparative transcriptomics reveal different genetic adaptations of biofilm formation in *Bacillus subtilis* isolate 1JN2 in response to Cd^2+^ treatment

**DOI:** 10.3389/fmicb.2022.1002482

**Published:** 2022-10-04

**Authors:** Wei Yang, Haixia Yan, Guanghui Dong, Zhengpeng Li, Chunhao Jiang, Dalu Gu, Dongdong Niu, Danni Zhou, Yuming Luo

**Affiliations:** ^1^Jiangsu Key Laboratory for Eco-Agricultural Biotechnology Around Hongze Lake, School of Life Science, Huaiyin Normal University, Huai’an, China; ^2^Jiangsu Collaborative Innovation Center of Regional Modern Agriculture & Environmental Protection, Huai’an, China; ^3^Agro-Tech Extension and Service Center, Huai’an, China; ^4^College of Plant Protection, Nanjing Agricultural University, Nanjing, China; ^5^Huaiyin Institute of Agricultural Sciences of Xuhuai Region in Jiangsu, Huaian Academy of Agricultural Sciences, Huai’an, China

**Keywords:** *Bacillus subtilis*, biofilm, cadmium, stress response, adaptation, transcriptomics analysis

## Abstract

Biofilm plays important roles in the life cycle of *Bacillus* species, such as promoting host and object surface colonization and resisting heavy metal stress. This study utilized transcriptomics to evaluate the impacts of cadmium on the components, morphology, and function of biofilms of *Bacillus subtilis* strain 1JN2. Under cadmium ion stress, the morphology of the *B. subtilis* 1JN2 biofilm was flattened, and its mobility increased. Moreover, differential gene expression analysis showed that the main regulator of biofilm formation, Spo0A, decreased in expression under cadmium ion stress, thereby inhibiting extracellular polysaccharide synthesis through the SinI/SinR two-component regulatory system and the AbrB pathway. Cadmium ion treatment also increased the SigD content significantly, thereby increasing the expression of the flagella encoding and assembly genes in the strain. This promoted poly-γ-glutamic acid production *via* the DegS/DegU two-component regulatory system and the conversion of biofilm extracellular polysaccharide to poly-γ-glutamic acid. This conferred cadmium stress tolerance in the strain. Additionally, the cadmium ion-mediated changes in the biofilm composition affected the colonization of the strain on the host plant root surface. Cadmium ions also induced surfactin synthesis. These findings illustrate the potential of *Bacillus* species as biocontrol strains that can mitigate plant pathogenic infections and heavy metal stress. The results also provide a basis for the screening of multifunctional biocontrol strains.

## Background

*Bacillus* is an important genus of plant growth-promoting rhizobacteria (PGPR) owing to its production of a variety of active metabolites and its strong resistance to environmental factors. Over the past years, more and more PGPRs have been used to assist host plants in resisting abiotic stress such as drought, cold, and heavy metal pollution (Yang et al., 2009).In this respect, biofilm plays a crucial role along with the occurrence of *Bacillus* in nature (Yang et al., 2018). A biofilm can protect an individual bacterium from environmental stress, such as antibiotic compounds (Høiby et al., 2010), and aid bacterial colonization of host plant roots (Bais et al., 2004; Beauregard et al., 2013). The latter is considered the first and most important defense technique that biocontrol agents use against soil-borne pathogens. Biofilm-enhanced mutants have been reported to exhibit enhanced biocontrol activities against *Ralstonia*, while biofilm-deletion mutants have reduced disease prevention capacity (Yang et al., 2018). Zhang et al. (2021) found that *Bacillus subtilis* B12 can secrete more surfactin under Cd stress to regulate biofilm formation to absorb more Cd. Kaçar et al. (2002) found that the enrichment of Cd^2+^ by *Bacillus* can reach 21.4% of its dry weight. However, there is no clear conclusion on which component of biofilm is involved in bacterial colonization and heavy metal adsorption.

Bacterial biofilms exist on the surface of bacteria, as aggregates formed by the extracellular polymers secreted by the bacteria themselves (Koo et al., 2017), including polysaccharides, proteins, nucleic acids, and lipids, which account for more than 90% of bacterial biofilms (Sutherland, 2001). The regulatory pathway for biofilm formation is well-defined in *B. subtilis* (Vlamakis et al., 2013). Briefly, the environmental signals (like plant root secretions) are perceived by sensory histidine kinases (such as KinC/KinD), which activate the global master regulator, Spo0A, through protein phosphorylation (López et al., 2009; Mcloon et al., 2011; Chen et al., 2012; Beauregard et al., 2013; Shemesh and Chai, 2013). The phosphorylated Spo0A (Spo0A-P) induces biofilm formation *via* two independent mechanisms that antagonize the two main repressors (SinR and AbrB) that inhibit biofilm formation (Hamon and Lazazzera, 2001; Chai et al., 2011). While in other species, several regulators were involved in biofilm formation as well as flagella assembly. For example, Zur is required for Zinc uptake and ensure the expression of flagella and biofilm formation in *Salmonella enterica* (Ammendola et al., 2016). Fur, the cytoplasmic ferric uptake regulator protein, ensure the mutant to form more mature biofilm than the parent in *Pseudomonas aeruginosa* (Banin et al., 2005). Like Fur, IdeR binds iron and then interacts with a specific sequence in the operator regions of iron-regulated genes to control their transcription coordinating iron homeostasis and morphological differentiation (Cheng et al., 2018). Another iron binding regulator, XibR in *Xanthomonas campestris*, not only can promote biofilm formation but also regulate the virulence of the pathogen (Pandey et al., 2016).

Abbreviations: EPS, extracellular polysaccharides; γ-PGA, poly-γ-glutamic acids; FPKM, fragments per kilobase of exon model per million mapped fragments.

Poly-γ-glutamic acid (γ-PGA), ranging from about 10 to 1,000 kDA in size, is an important component of *B. subtilis* biofilm matrix (Morikawa et al., 2006; Poo et al., 2010; Ogunleye et al., 2015). Its biosynthesis in *B. subtilis* is regulated by the conserved operon *pgsB-pgsC-pgsA-pgsE* (previously named *ywsC-ywtA-ywtB-ywtC*) (Ashiuchi and Misono, 2002). These genes are highly conserved across different *Bacillus* species, including *B. cereus* and *B. anthracis* (Makino et al., 1989; Stanley and Lazazzera, 2005). In *B. subtilis*, the *pgs* operon is regulated by two cascades (ComA-ComP and DegS-DegU) of the two-component system (Stanley and Lazazzera, 2005). It has been shown that regulatory mutants of *B. subtilis* 168 degU and *B. subtilis* 168 ccpA are more resistant to the addition of divalent metal ions (Ca^2+^, Mg^2+^, Mn^2+^, and Zn^2+^) than wild-type *B*. *subtilis* (Dinh et al., 2019).

Furthermore, γ-PGA biosynthesis may interact with the regulatory pathway of extracellular polysaccharide (EPS) production. Yu et al. (2016) reported that down-regulated expression of *epsD* and *yqxM*, which are responsible for biofilm formation, have minimal effects on the expression of the γ-PGA synthesis gene *ywtB*. In contrast, overproduction of γ-PGA in *B. amyloliquefaciens* C06 and its *epsA* and *tasA* mutants resulted in reduced EPS and TasA levels (Liu et al., 2010). Moreover, KinC and KinD negatively regulate γ-PGA production in *B. subtilis* by activating Spo0A, which down-regulates AbrB activities. Thus, this pathway positively regulates biofilm formation but negatively controls γ-PGA production, suggesting the existence of a noteworthy switch-like mechanism of EPS and γ-PGA production. In contrast, the reverse regulatory pathway mediated by the DegS-DegU two-component system positively regulates γ-PGA production but strongly inhibits biofilm matrix genes (Yu et al., 2016).

Many environmental factors affect the formation of bacterial biofilms. Ma and Sun (2021) found that the expression levels of many biofilm formation-related genes, including those that encode proteins involved in flagellar assembly, signal transduction, bacterial secretion, and the TonB-dependent transfer system, were significantly upregulated when facing Cd stress, indicating their critical roles in determining bacterial biofilm formation and enhancing Cd resistance. Li et al. (2020) also found that the upregulation of genes related to biofilm formation in *Pseudomonas chenduensis* strain MBR (i.e., *algK, algX*, and *alg44*) enabled Cd absorption and contributed to Cd bioremediation. However, Alviz-Gazitua et al. (2019) found that inhibition of the initiation of biofilm formation and EPS production by *Cupriavidus metallidurans* CH34 is positively correlated with cadmium concentration.

Poly-γ-glutamic acid plays different roles in various *B. subtilis* isolates. Deleting *ywsAB* and *ywsC*, homologs of *pgsBCA*, resulted in weak biofilm formation in *B. subtilis* JH642; however, there were no differences in biofilm formation between *pgsBCA* mutant and wild-type *B. subtilis* 3,610 (Stanley and Lazazzera, 2005; Branda et al., 2006). Moreover, γ-PGA has been proven essential for *B. amyloliquefaciens* C06 and *B. subtilis* isolate colonization of apple (Liu et al., 2010) and tomato root surfaces (Yu et al., 2016), respectively. Similarly, EPS, another important component of biofilms, has also been shown to help biocontrol strains achieve effective colonization (Zhu et al., 2020).

Although the regulatory pathways of EPS production and γ-PGA biosynthesis have been well studied, the cause of their production shift is still unclear. It is also unknown whether changes between these two biofilm components might affect other activities, such as colonization and biocontrol ability of these strains. *B. subtilis* strain 1JN2 was isolated in our previous work and found to be an efficient biocontrol agent against *Ralstonia* wilt (Yang et al., 2012). In addition, with the acceleration of industrialization and the continuous growth of population, heavy metal pollution has become a global problem (Chen et al., 2015). Among them, cadmium is the main culprit threatening agricultural production. According to the latest “National Soil Pollution Survey Bulletin” (2014), cadmium ranks first among inorganic pollutants and the area of cadmium contaminated farmland has exceeded 2.0 × 10^4^ hm2. Owing to the seriousness of cadmium pollution in China, we tested the resistance of this strain to cadmium ion contamination as well as its ability to protect host plants; although the biofilm morphology of this strain changed after cadmium treatment, it did not affect its control effect on *Ralstonia* wilt (Yang et al., 2015). Comparative transcriptomics was used in the present study to evaluate the impacts of cadmium on the components, morphology, and function of biofilms of a *B. subtilis* strain 1JN2. It was demonstrated that high concentrations of Cd^2+^ induced γ-PGA biosynthesis and inhibited EPS production in *B. subtilis* 1JN2. The shift between EPS and γ-PGA production enabled the strains to adapt to heavy metal contamination and persistently control host plant diseases.

## Materials and methods

### Strains, reagents, and media conditions

We used a wild *B. subtilis* strain 1JN2, previously isolated by Yang et al. (2018) and deposited in the China General Microbiological Culture Collection Center (CGMCC, NO 9759), for the present experiments. Competent 1JN2 strains containing chloramphenicol resistance were prepared and then electrotransformed with a green fluorescent plasmid (pGFP 4,412) for fluorescent microscopy using the method described by Xue (2008). All strains were routinely grown in Luria-Bertani (LB) broth (10 g/l of tryptone, 5 g/l of yeast extract, and 5/L g of NaCl) or LB medium with 1.5% agar. Conversely, biofilm formation was conducted using LBGM medium (LB + 1% (*v*/*v*) glycerol +0.1 mM MnSO_4_) (Shemesh and Chai, 2013).

### Biofilm formation assay

A stock solution containing 1 M Cd^2+^ was prepared using cadmium sulfate (3CdSO_4_-8H_2_O) and sterilized by filtration through a 0.22-μm bacterial filter followed by storage at 4°C.

To analyze the impacts of Cd^2+^ on biofilm formation, five μL of 1JN2 suspension (10^6^ CFU/ml) was distributed as drops on each LBGM agar plate containing different Cd^2+^ concentrations, including 0, 1, 2, 3, 4, and 5 mM. Each concentration was repeated three times. The plates were incubated at 30°C for 72 h, and then the diameter and the height of each biofilm were measured by Vernier caliper.

### Tomato root colonization by *Bacillus subtilis* 1JN2

Three treatment categories, (A) water, (B) *B. subtilis* 1JN2, and (C) *B. subtilis* 1JN2 + Cd^2+^, were used to detect the colonization ability of tomato plants (obtained from the Shanghai Cooperation 903 seed market, Shanghai, China) by *B. subtilis* 1JN2 after cadmium ion treatment. Each treatment was conducted in three replicates with 20 plants per replicate. Tomato plants with three to four true leaves were transferred from seedling trays into experimental pots (10 cm height × 10 cm diameter) filled with 0.5 kg of nutrient soil. Each pot was supplied with 20 ml of *B. subtilis* 1JN2 culture (10^7^ CFU/ml) 7 days after transplantation. Thereafter, pots in the treatment C category were supplemented with 20 ml of Cd^2+^ solution (6 mM) 14 days after transplantation. Greenhouse growth conditions included a temperature of 30°C with 16/8 h of light/dark photoperiod.

After treatment, the plant roots were collected 21 days post-transplantation and divided into two parts. The first part of the root sample was immersed in sterile water, and then, its population of 1JN2 was measured by dilution spreading on chloramphenicol-containing plates. The second part of the root sample was analyzed using a ZEISS LSM 700 confocal microscope (Carl Zeiss AG, Oberkochen, Germany) after fixation. The colonization ability of 1JN2 was evaluated under treatments B and C by using a fluorescent analysis described by Yang et al. (2018). GFP signal was selected *via* a Smart Setup, and the excitation and emission wavelengths were 488 nm and 493–550 nm, respectively.

### Collection of Cd^2+^-treated bacterial cells

We inoculated 0.5 ml of 1JN2 suspension (10^6^ CFU/ml) into 50 ml of LB broth supplemented with 3 mM Cd^2+^ and incubated the cells at 30°C for 24 h. The inoculation was conducted in twelve replicates, among which three replicates each were used for bacterial cells collection at 6, 12, 18, and 24 h after inoculation. Bacterial cells were also collected from a blank LB broth (control) inoculated in triplicate without Cd^2+^ at 24 h post-inoculation. The cell collection was conducted by centrifugation at 5000 rpm for 5 min and then stored in liquid nitrogen prior to RNA extraction. The obtained supernatants were used for EPS and γ-PGA content detection.

### EPS and γ-PGA content detection following 1JN2 treatment with Cd^2+^

The same treatment described above was conducted for the purpose of EPS and **γ-**PGA content detection, and each treatment consisted of three replicates. For EPS detection, 2 ml of the supernatant was transferred into test tubes and 6 ml of anthrone reagent was added to each test tube. The solutions were then properly mixed and heated in boiling water for 15 min, followed by cooling in ice water for 15 min. The absorbance of each sample was measured at a wavelength of 625 nm, and their sugar contents were determined using a glucose standard curve.

Meanwhile, for γ-PGA detection, 3 ml of methylene blue (color developing solution; 10 mg/l) was added into test tubes containing 3 ml of the supernatants and vortexed for 5 min at 25°C. A wavelength of 664 nm was used for measuring the absorbance of each sample, and the γ-PGA content of the samples was determined based on a γ-PGA standard curve.

### RNA extraction and preparation

Total RNA was isolated from the collected cells using Tiangen reagent (Tiangen, Beijing, China) and subsequently purified using a QIAGEN RNeasy MINI kit (QIAGEN, Hilden, Germany) according to the manufacturers’ instructions. The RNA quality and quantity were determined at a 260 nm wavelength *via* UV spectroscopy (NanoPhotometer® spectrophotometer; IMPLEN, Westlake Village, CA, United States) and analyzed on an RNA 6000 Nano Labchip using 2100 bioanalyzer (Agilent Technologies, Santa Clara, CA, United States).

### Library preparation for strand-specific transcriptome sequencing

Library preparation was conducted at Genepioneer Biotechnologies Co., Ltd., Nanjing, China, using 3 μg of RNA from each sample based on a protocol described by Yang et al. (2019). Illumina sequencing libraries were generated using a NEBNext^®^ Ultra™ Directional RNA Library Prep Kit (NEB, Ipswich, MA, United States). Briefly, a random hexamer primer and M-MuLV Reverse Transcriptase (RNase H–) were used for first-strand cDNA synthesis, while the second cDNA strand was synthesized using DNA polymerase I and RNase H. NEBNext Adaptors (NEB) with hairpin loop structure were ligated to the cDNA fragments after adenylation to prepare for hybridization. The library fragments were then purified using the AMPure XP system (Beckman Coulter, Beverly, MA, United States), and their quality was assessed on the Agilent Bioanalyzer 2100 system (Lei et al., 2018).

### Clustering and sequencing

The samples were index-coded and clustered on a cBot Cluster Generation System using TruSeq PE Cluster Kit v3-cBot-HS (Illumina, San Diego, CA, United States), according to the manufacturer’s instructions. An Illumina HiSeq platform (Cloud Health, Nanjing, China)^1^ was then used for library sequencing and paired-end read generation.

### Data analysis

The initial raw data obtained in fastq format were processed using in-house Perl scripts. Subsequently, HISAT2 2.0.5 (Zhang et al., 2016) was used for reference genome indexing and aligning clean reads to the reference genome. The reads mapped to each gene were counted using String Tie (Pertea et al., 2016). The fragments per kilobase of exon model per million mapped fragments (FPKM) value of each gene was then calculated based on their length and the read counts mapped to the gene (Lei et al., 2018). Differential expression analysis of the groups was performed using the DESeq R package (1.18.0), while the GOseq R package (with corrected gene length bias) was utilized for Gene Ontology (GO) enrichment analysis of the differentially expressed genes. BLAST software was used to test the statistical significance of enrichment of the differentially expressed genes in Kyoto Encyclopedia of Genes and Genomes (KEGG) pathways (Li et al., 2016).

### Quantitative real-time PCR analysis

The expression levels of five selected genes of *B. subtilis* 1JN2 cultured under similar conditions were validated by real-time RT-PCR using an iCyclerMyiQ Real-Time PCR System (Bio-Rad, Hercules, CA, United States). The PCR conditions were set as follows: initial denaturation at 95°C for 2 min, followed by 40 cycles of denaturation at 95°C for 10 s, annealing at 65°C for 15 s and extension at 72°C for 20 s. Melting curve analysis of the amplicons was performed at the end of each PCR run to ensure that unique products had been amplified.

## Results

### Cd^2+^ treatment changed biofilm formation by *Bacillus subtilis* 1JN2

**Figure 1:**
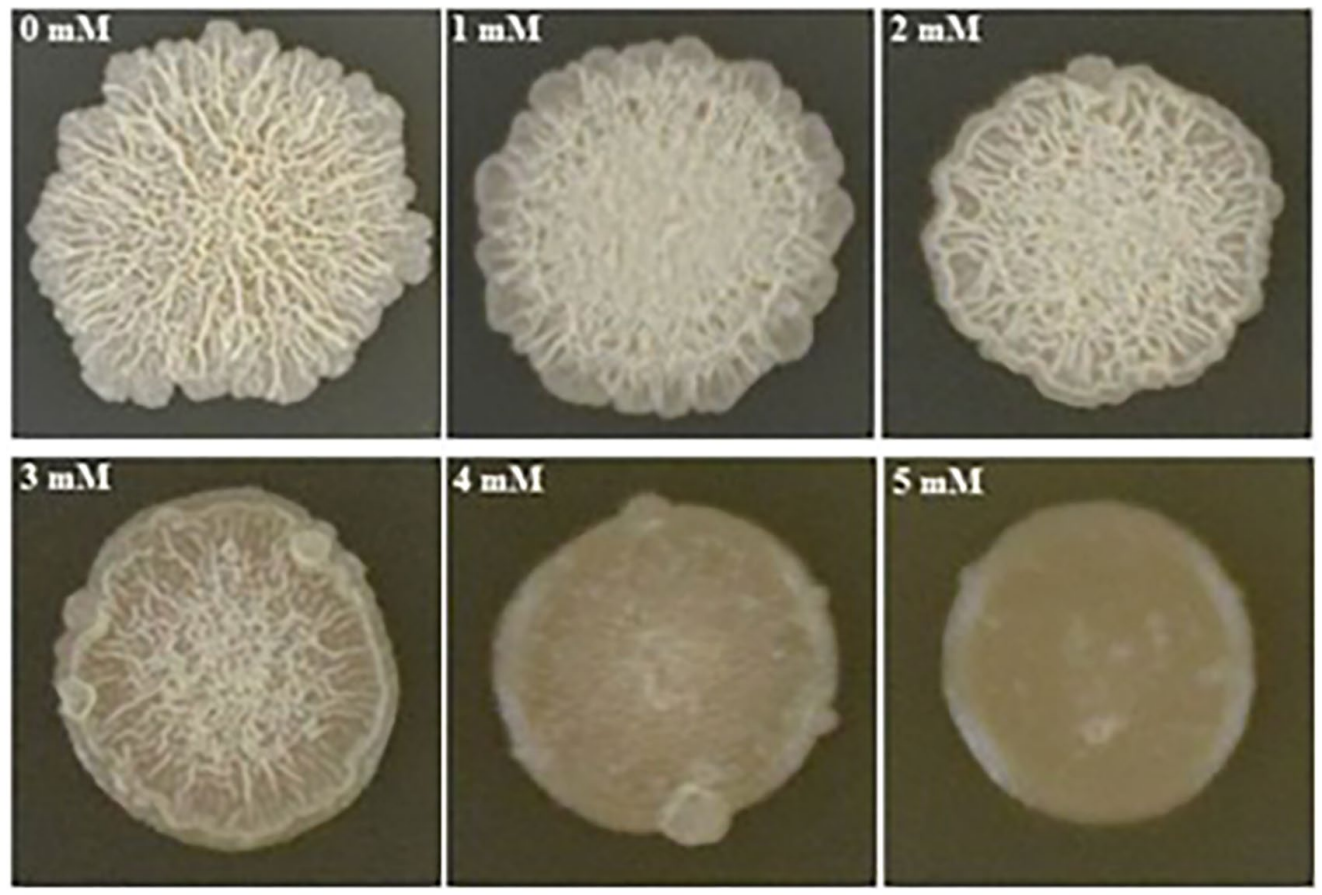
Impacts of Cd2^+^ on the biofilm colonies of *B. subtilis* 1JN2. 5 ul of 1JN2 suspension (10^6^ CFU/ml) was spotted on the surface of LBGM agar plate with a Cd2^+^ gradient of 0, 1, 2, 3, 4 and 5 mM, the picture was taken at 72 h post incubation at 30°C.

We found that biofilm formation by *B. subtilis* 1JN2 varied with the Cd^2+^ concentration (Figure 1). In general, as cadmium ion concentration increased, the biofilm gradually became smaller and the surface gradually became smoother. Increasing Cd^2+^ concentration from 0 to 2 mM moderately inhibited biofilm formation by 1JN2, but the biofilm morphology did not change markedly; however, Cd^2+^ concentrations of 3 mM or higher significantly inhibited biofilm formation by 1JN2 (Supplementary Table 1), and not only the diameter but also the height decreased significantly. In contrast to biofilm formation, there was no significant difference in the growth of 1JN2 under a Cd^2+^ concentration of 0 to 2 mM Cd^2+^ (Yang et al., 2018).

The biofilm forms were also affected by Cd^2+^ treatment. Biofilm surfaces of the Cd^2+^-treated cells were complanate compared to the convex surfaces of the group without Cd^2+^. Moreover, the viscosity of the biofilms also increased with the Cd^2+^ concentration.

### Cd^2+^ treatment influenced tomato root colonization by *Bacillus subtilis* 1JN2

**Figure 2:**
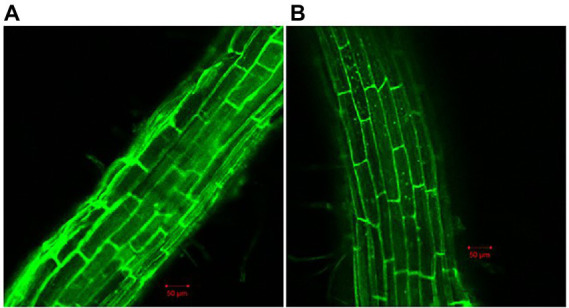
Confocal microscope detection of *B. subtilis* 1JN2 on the root of tomato with **(B)** or without **(A)** Cd2^+^ treatment. Fluorescent analysis was carried out using ZEISS LSM 700. GFP was selected in Smart Setup, the excitation wavelength is 488 nm, and the emission wavelength is 493–550 nm.

Because biofilm formation is an important step in root colonization, it was also imperative to evaluate the effect of Cd^2+^ on the root colonization ability of *B. subtilis* 1JN2. A GFP-tagged 1JN2 was constructed for the root colonization experiment in the greenhouse. The population of *B. subtilis* 1JN2, which colonized the tomato roots, decreased with Cd^2+^ treatment (Supplementary Figure 1), indicating that biofilm inhibition caused by Cd^2+^ affected the colonization ability of 1JN2. However, as shown in Figure 2, the Cd^2+^ treatment only affected the colonization ability on the surface of tomato roots. A large number of bacterial cells entered the inside of tomato roots, and the difference was not significant compared with the treatment group without cadmium ions (Figure 2). In addition, Compared with the control group without cadmium ions, we can see that the strain could colonized on the surface of tomato roots in the control group, so the effect of chelating cadmium ions by other components in the rhizosphere can be excluded.

### RNA sequencing and identification of differentially expressed genes

Cell samples were collected at four time points after Cd^2+^ treatment; the samples collected at 6, 12, 18, and 24 h were denoted S2, S3, S4, and S5, respectively, while the blank control without Cd^2+^ was designated S1. The sequence data are summarized in Table 1. More than 93% of the clean reads from each sample mapped to the reference genome, indicating that the obtained transcriptome data were suitable for further analysis. The raw data were deposited to the NCBI SRA database under accession number PRJNA646606.^2^

**Table 1:** Summary of RNA-seq data and the reads mapped to the *Bacillus subtilis* 1JN2 genome.

Sample	Read number	Base number	GC content (%)	% ≥ Q30 (%)
S1-1	7,996,483	2,398,944,900	45.09	95.83
S1-2	9,237,500	2,771,250,000	45.08	96.31
S1-3	10,721,600	3,216,480,000	44.98	94.94
S2-1	10,667,725	3,200,317,500	44.49	95.43
S2-2	9,264,843	2,779,452,900	44.54	93.99
S2-3	9,528,611	2,858,583,300	44.29	94.29
S3-1	10,855,623	3,256,686,900	44.37	94.71
S3-2	8,856,524	2,656,957,200	44.13	94.52
S3-3	9,460,244	2,838,073,200	44.43	93.75
S4-1	11,315,033	3,394,509,900	44.59	94.51
S4-2	10,009,680	3,002,904,000	44.66	94.13
S4-3	9,704,248	2,911,274,400	44.35	93.06
S5-1	8,895,735	2,668,720,500	44.68	90.67
S5-2	9,932,927	2,979,878,100	44.69	93.12
S5-3	9,358,331	2,807,499,300	44.71	94.85

**Figure 3:**
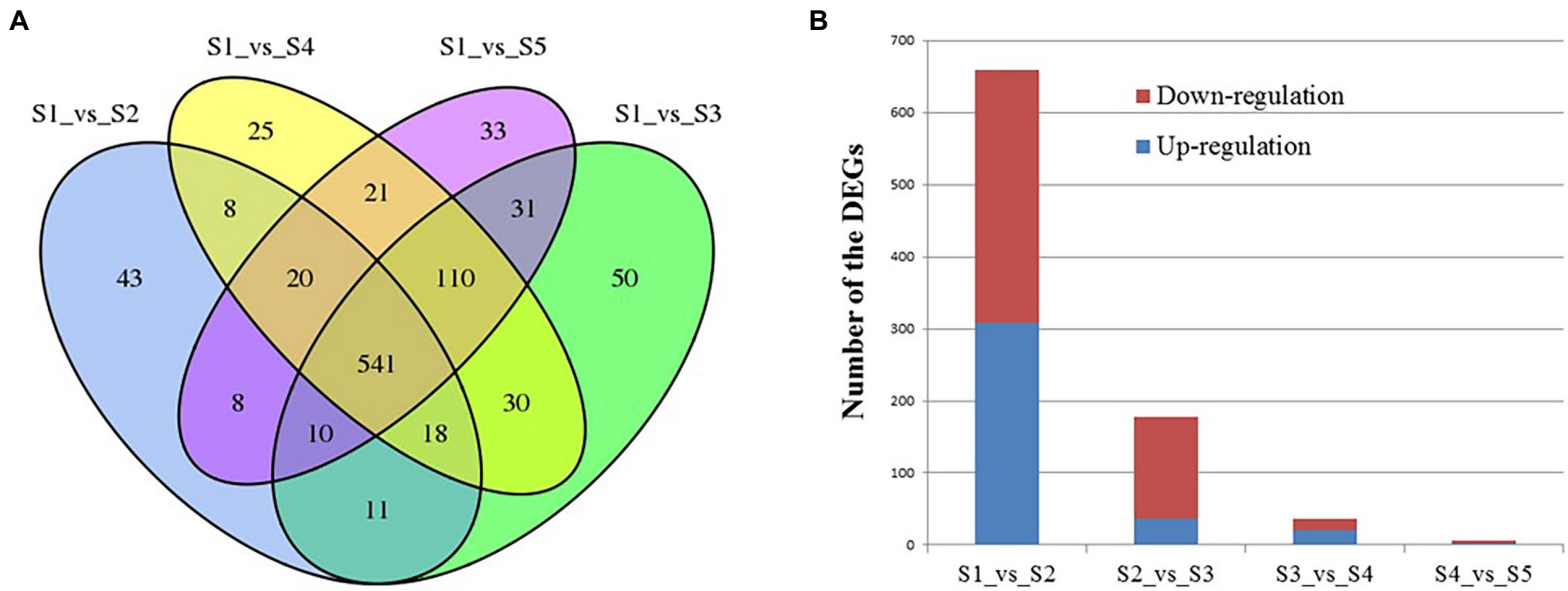
Analysis of the differentially expressed genes of *B. subtilis* 1JN2 after Cd2^+^ treatment. **(A)** Venn diagram analysis, **(B)** numbers of DEGs between time points. Samples collected at 6 h, 12 h, 18 h, and 24 h were named as S2, S3, S4, S5 and the blank control that without Cd2^+^ was named as S1. Comparison between S1 and S2 means the DEGs between the two samples, and so on.

The numbers of differentially expressed genes (DEGs) between the various time points after Cd^2+^ treatment of *B. subtilis* 1JN2 were visualized as a Venn diagram (Figure 3A). The number of DEGs between S1 and S2 decreased with the prolongation of treatment time. There were 659 DEGs between S1 and S2, among which 308 were up-regulated, and 351 were down-regulated. However, the number of DEGs between S2 and S3, S3 and S4, and S4 and S5 were 178, 36 and 6, respectively (Figure 3B). This implies that the cells undergo an important adjustment phase during the initial period of exposure to Cd^2+^ and later acquire resistance to Cd^2+^ stress. The same trend was observed in the principal component analysis results (Supplementary Figure 2), which showed greater similarity among S3, S4, and S5, consistent with a lack of significant differences in their gene expression.

### Cell mobility-related genes changed significantly after Cd^2+^ treatment

**Figure 4:**
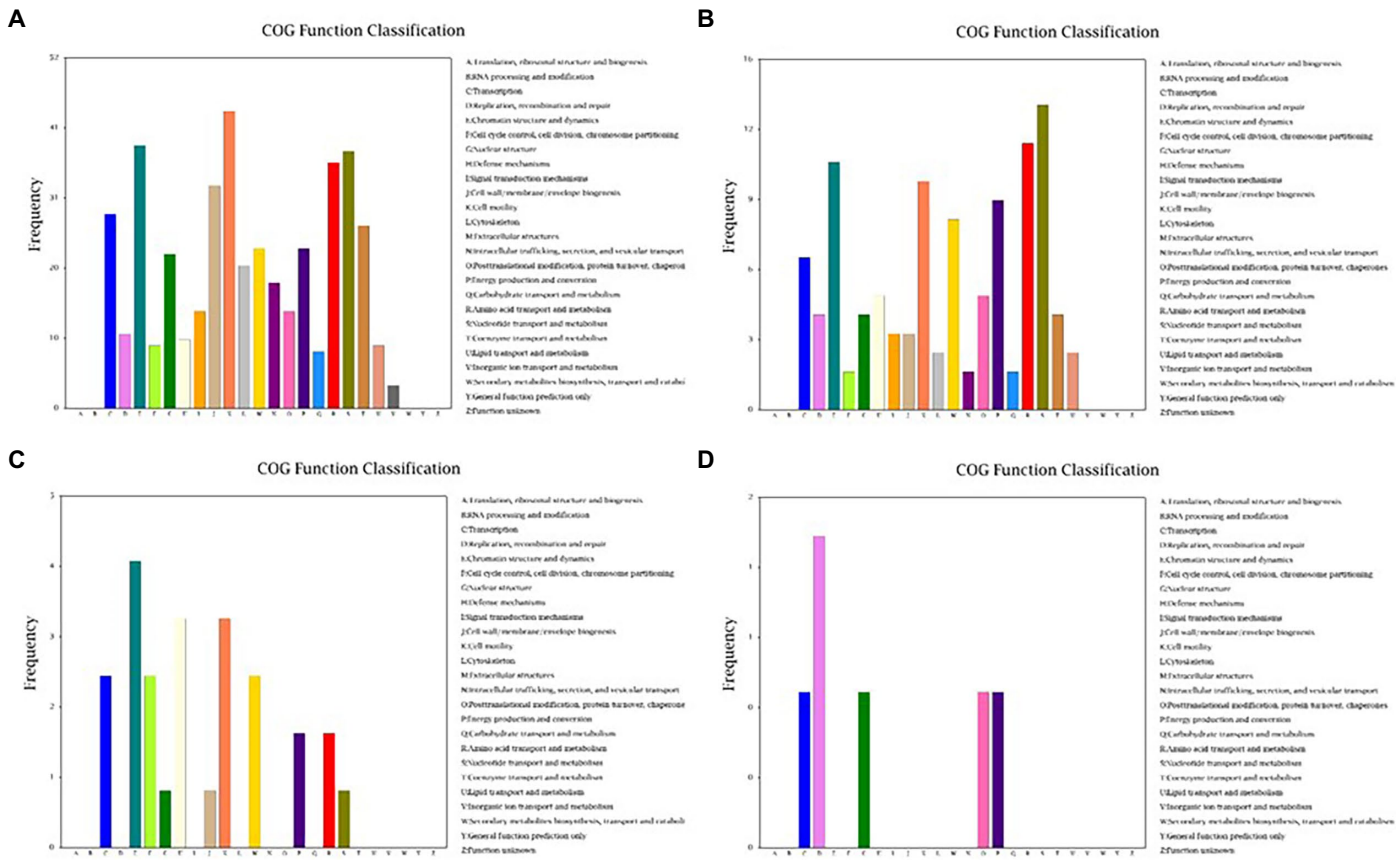
Cluster of Orthologous Groups of Proteins (COG) function classification of the DEGs of *B. subtilis* 1JN2 after Cd2^+^ treatment. **(A–D)** Means the comparison of DEGs according to COG function classification between S1 and S2, S2 and S3, S3 and S4, S4 and S5 separately.

Because Cd^2+^ treatment modified the morphological features of strain 1JN2 biofilm, we checked whether it could also affect the mobility of the strain. The Cluster of Orthologous Groups of Proteins (COG) database is constructed based on the phylogenetic relationship between bacteria, archaea and eukaryotes. Applying BLAST to the database allows for orthologous classification of gene products. The COG database BLAST results showed that the most significant changes in gene products of the strain after exposure to Cd^2+^ were related to cell mobility, followed by chromatin structure and dynamics, amino acid and nucleotide transport, and metabolism (Figure 4). Moreover, a comparison between S1 and S2 indicated that the strain could perceive and respond to Cd^2+^ stress after some period of exposure *via* regulatory metabolism. These results are consistent with the observed biofilm morphological changes of the strain.

### Expression of flagella synthesis-and assembly-related genes increased in strain 1JN2 after Cd^2+^ treatment

**Figure 5:**
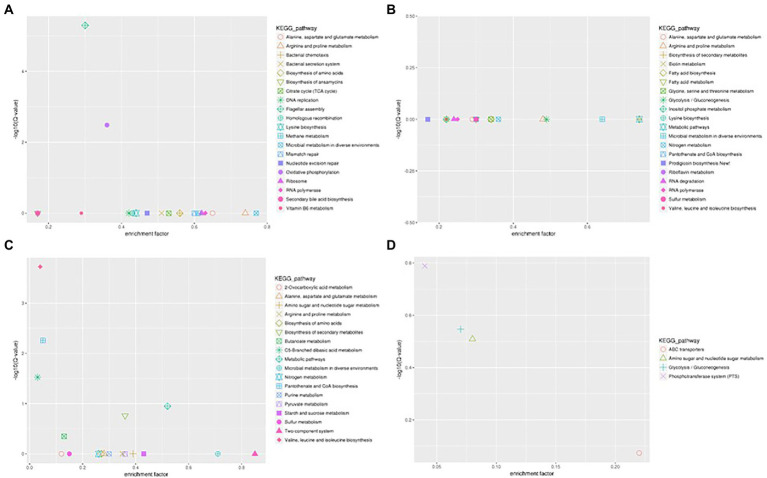
Scatter plot of Kyoto Encyclopedia of Genes and Genomes (KEGG) pathway enrichment of the DEGs of *B. subtilis* 1JN2 after Cd2^+^ treatment. **(A–D)** Means the comparison of DEGs according to KEGG pathway enrichment between S1 and S2, S2 and S3, S3 and S4, S4 and S5 separately.

**Figure 6:**
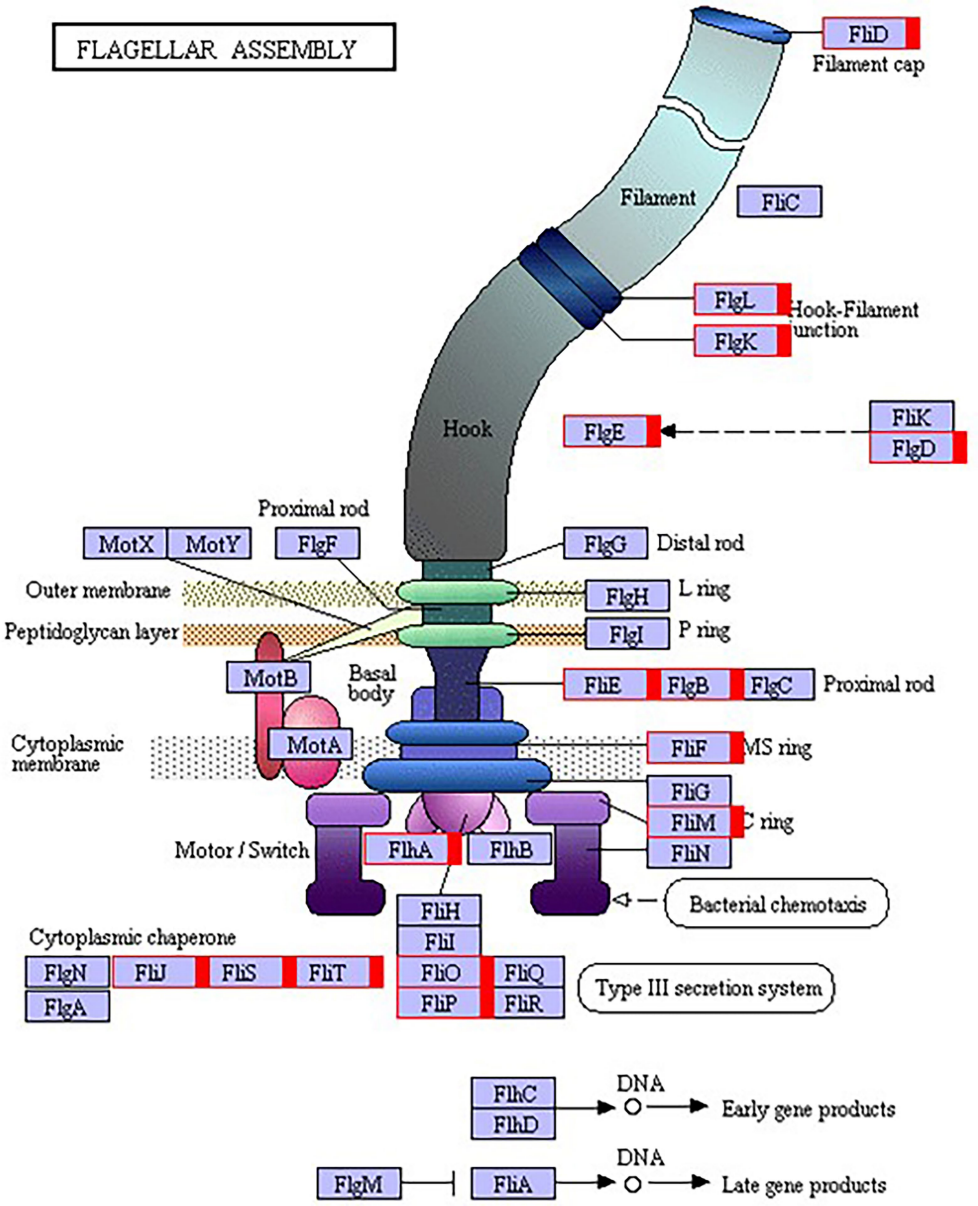
Kyoto Encyclopedia of Genes and Genomes (KEGG) pathway annotation of the genes involved in flagella encoding and assembly of *B. subtilis* 1JN2 after Cd2^+^ treatment. Genes markered with red indicate up-regulation.

The flagellum is the most important motor organ, which plays an important role in the mobility and chemotaxis of bacterial cells. Owing to the observed significant effects of Cd^2+^ on the strain’s mobility, we assessed its effects on flagella synthesis and assembly genes. KEGG is a database for systematic gene function analysis and genomic information. Significant pathway enrichment can indicate the main biochemical, metabolic, or signal transduction pathways associated with differentially expressed genes. According to KEGG analysis, flagella synthesis and assembly genes were significantly enhanced relative to the control group 12 h after Cd^2+^ treatment (Figure 5). The differential genes at the later sampling time points were mainly involved in metabolism-related reactions, such as glycolysis/gluconeogenesis and biosynthesis of valine, leucine, isoleucine, pantothenate, and CoA. Figure 6 also illustrates similar results.

### Effect of Cd^2+^ on the extracellular secretions of strain 1JN2

**Figure 7:**
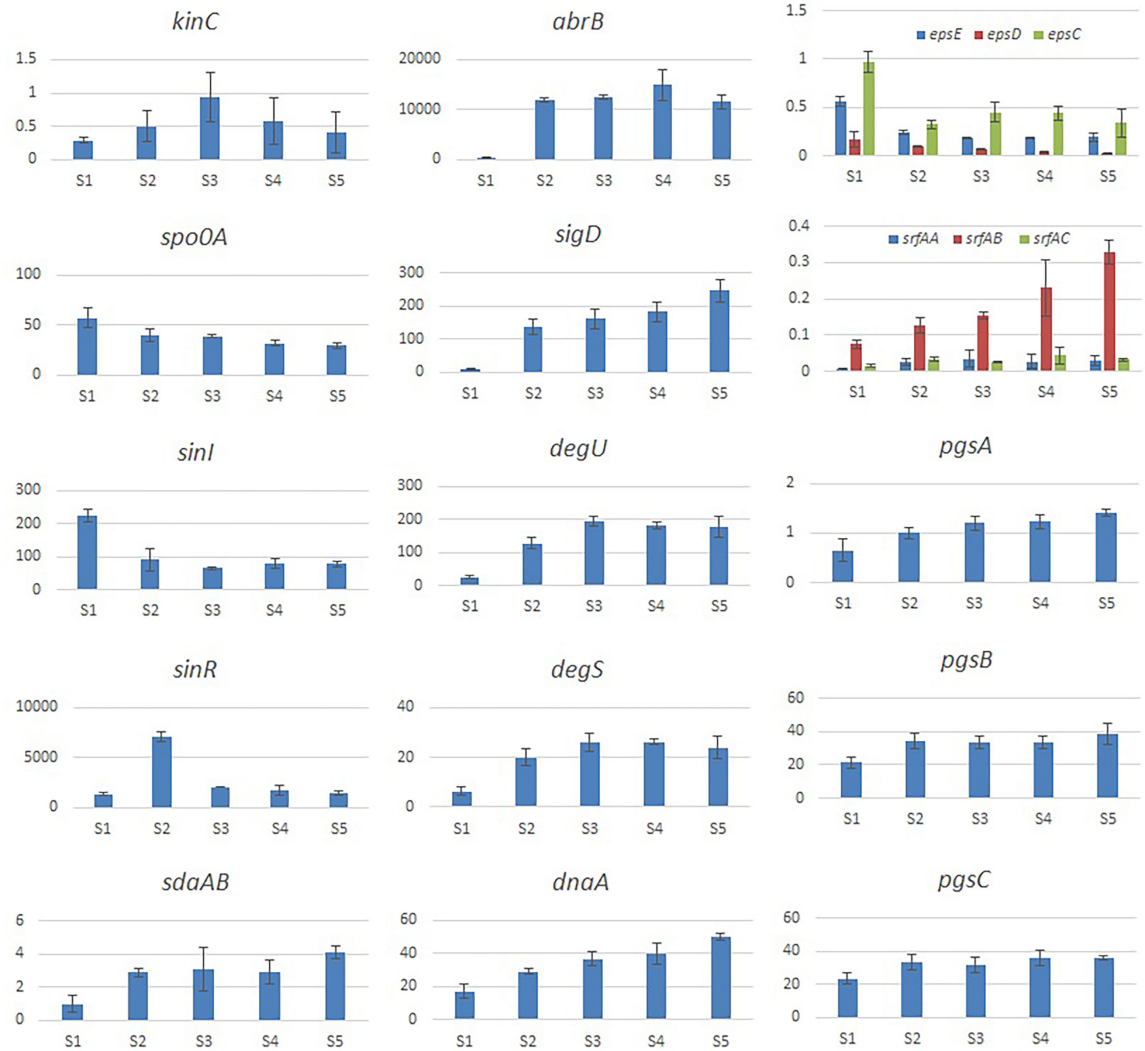
Relative expression level of the genes involved in extracellular secretion and regulation of *B. subtilis* 1JN2 after Cd2^+^ treatment. The histogram was based on the expression level in FPKM (Fragments per Kilobase of Transcript per Million Fragments Mapped) value.

**Figure 8:**
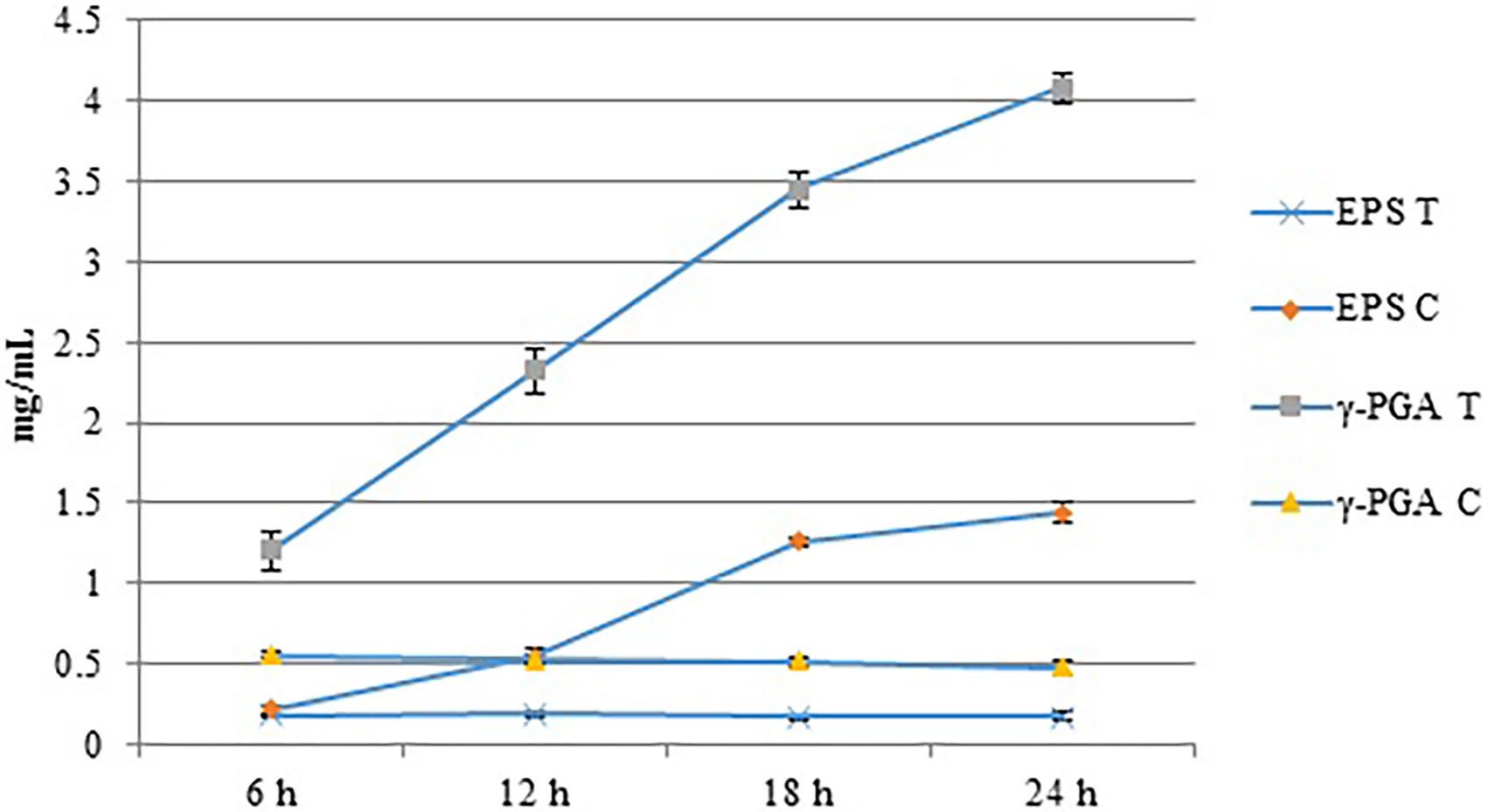
The content of EPS and γ-PGA of the strain 1JN2 after Cd^2+^ treatment. 3 mM Cd^2+^ was added in the broth and the supernatants at 6, 12, 18 and 24 h after inoculation were used to detect the content of EPS and γ-PGA. T means treated with Cd^2+^ and C means control without Cd^2+^.

We evaluated the effects of Cd^2+^ stress on the extracellular secretions (polysaccharides and proteins) of strain 1JN2 and found that the extracellular protein-related genes (*tapA-sipW-tasA*) were not expressed in the strain. However, the expression of EPS-encoding genes (*epsA-O*) decreased significantly after Cd^2+^ treatment, indicating that Cd^2+^ reduces polysaccharides levels in *B. subtilis* 1JN2 biofilm (Figure 7). According to Yu et al. (2016), γ-PGA plays an important role in polymorphism of *B. subtilis* biofilm; therefore, we assessed the expression of the γ-PGA-encoding genes in this study. Moreover, the genes encoding γ-PGA (*pgsB-pgsC-pgsA*) were highly expressed after Cd^2+^ treatment, but there was no significant difference in the expression across the last four sampling time points. This indicated that changes in the extracellular secretions mainly occurred within a certain period after 1JN2 exposure to Cd^2+^ treatment. Similar results were obtained by comparing the changes in EPS and γ-PGA levels at different time points after Cd^2+^ treatment. The EPS levels remained unchanged at very low concentrations, while γ-PGA content increased significantly compared to the control group Figure 8).

Additionally, studies have reported that surfactant secretion by *Bacillus* increases after exposure to cations; thus, we evaluated the expression of surfactin-encoding genes. The expression levels of the genes related to the surfactin-related genes (*srfAA-srfAB-srfAC*) were also significantly increased following Cd^2+^ treatment (Figure 7).

### Effect of Cd^2+^ on the regulation pathway of 1JN2 biofilm formation

According to related reports, multiple histidine kinases (KinA, KinB, KinC, KinD, and KinE) mainly sense exogenous environmental signals and collectively act on Spo0A either directly through protein phosphorylation or indirectly *via* a phospho-relay (Mcloon et al., 2011). We examined the expression of the regulatory factors of the biofilm formation pathway of 1JN2 and found that the expression of *kinC* significantly increased after exposure to Cd^2+^, reaching its highest level at 24 h (Figure 7). This shows that KinC plays an important role in detecting the change in exogenous ion signals. It has been reported that KinC induces low levels of Spo0A-P in response to potassium cation evacuation caused by surfactin-generated pores in the *B. subtilis* membrane (López et al., 2009). However, the main regulatory factor showed a downward trend. Therefore, increased kinC levels reduced the expression of Spo0A after exposure to Cd^2+^ (Figure 7). To explain this, we also examined the trends in Sda-and DnaA-encoding genes following Cd^2+^ treatment. Sda is a critical protein that controls the sporulation or biofilm states of bacterial cells. It can prevent the transfer of phosphate groups from histidine kinase to Spo0F, thereby blocking or delaying Spo0A activity (Whitten et al., 2007; Yan et al., 2016). The replication initiation protein, DnaA, activates *sda*, which effectively prevents phosphate group accumulation and activates Spo0A, thereby preventing cells from premature sporulation (Burkholder et al., 2001). Sda expression and activity are reduced when the cell enters the stationary phase, thus reversing the Spo0A and proteolytic activities of the existing Sda protein (Ruvolo et al., 2006). Accordingly, the increased expression levels of *dnaA* and *sdaAB* after exposure to Cd^2+^may have led to the changes in Spo0A. Meanwhile, the two-component regulatory system SinI/SinR limited the production of extracellular polysaccharides, similar to the effects of increased expression of AbrB-encoding genes after Cd^2+^ treatment.

We also evaluated the changes in the γ-PGA regulatory factors and found that SigD significantly increased after Cd^2+^ treatment. This activated the expression of flagella encoding and assembly genes by regulating γ-PGA production through another two-component regulatory system called DegU/S. Expression of the components of this regulatory pathway (*sigD*, *degS*, and *degU*) increased consistently, resulting in (n of flagella encwhich in turn likely enhanced the mobility of the strain. The changes in the expression levels of γ-PGA and EPS are consistent with the previously reported switch-like mechanism (Yu et al., 2016).

### Real-time PCR validation of the selected DEGs

**Figure 9:**
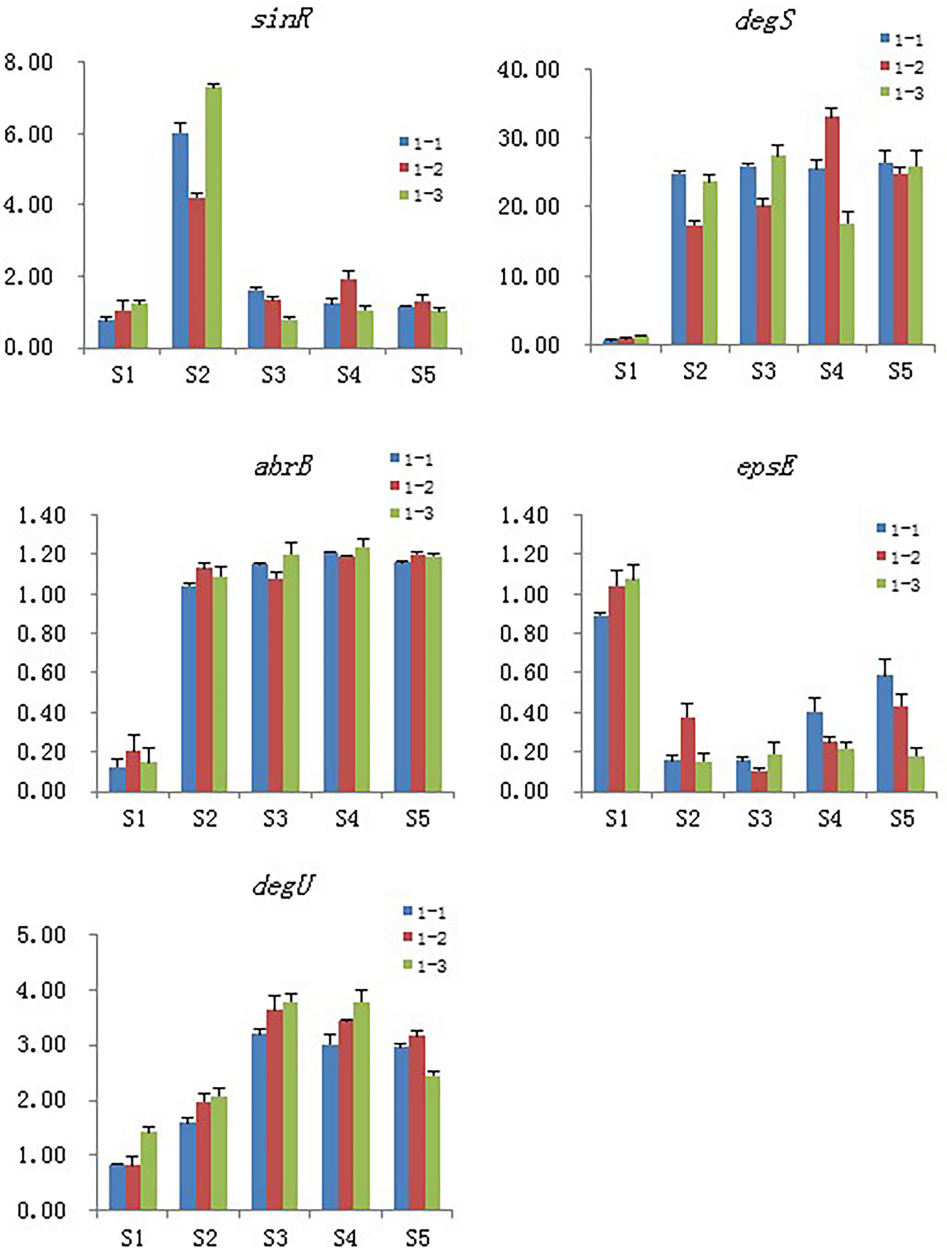
Validation of the selected DEGs by real-time PCR. Samples collected at 6 h, 12 h, 18 h, and 24 h were named as S2, S3, S4, S5 and the blank control that without Cd2^+^ was named as S1, 1–1, 1–2 and 1–3 means the three replicates of each sample.

Five genes were selected for qPCR assay to confirm the reproducibility and accuracy of the transcriptome data. We found only minor differences between the two data sets from RNA-seq and RT-PCR analyses (Figure 9).

## Discussion

Biofilms play important roles in the life cycle of *Bacillus* (Branda et al., 2001; Vlamakis et al., 2013), such as protection from antibiotics (Høiby et al., 2010) and adherence to the surface of objects. *Bacillus* is a preferable biocontrol agent of plant pathogens because of its strong survivability; therefore, various *Bacillus* species have been screened from the laboratory and applied in the field (Chen et al., 2012). Colonization is a very important factor in biocontrol efficacy since the level of colonization directly affects the inhibition capacity against plant pathogens of biocontrol agents (Chen et al., 2013). Various factors such as light, pH, and metal ions affect the colonization ability of biocontrol strains in the field. These factors also represent bottlenecks limiting the application of laboratory-generated biocontrol strains in the field.

Among the main components of *Bacillus* biofilm, both EPS and γ-PGA were reported to help the strain to colonize the host roots, thus inhibiting plant pathogens effectively (Yu et al., 2016). Wild-type *Bacillus* species exhibit different biofilm morphologies owing to differences in their extracellular secretions. Moreover, the role of these components in biofilm formation and morphological changes has been demonstrated using deletion mutants of different extracellular secretory components (Yan et al., 2016; Yu et al., 2016). Our previous work demonstrated that Cd^2+^ can change the biofilm morphology of *B. subtilis* 1JN2 (Yang et al., 2015). High Cd^2+^ concentrations increased the viscosity and flattened the surface of the 1JN2 biofilm, showing that exogenous heavy metal ions can affect the composition of the extracellular secretion of the strain. Accordingly, it is necessary to evaluate the mechanisms involved in the extracellular component change and determine whether the biological functions of the strain are affected by the change.

*B. subtilis* 1JN2 has proven to be a significant biocontrol agent against *Ralstonia via* host colonization (Yang et al., 2012). In a follow-up study, we found that the strain can effectively adsorb cadmium ions in the environment to relieve the stress to host plants (Yang et al., 2015). After cadmium ion treatment, the biofilm morphology of the strain changed significantly, but its biocontrol effect on bacterial wilt did not change significantly. Thus,we assessed whether the biofilm morphological changes caused by Cd^2+^ impact the colonization ability of this strain. The results show that the change in extracellular components caused by Cd^2+^ treatment reduced tomato root surface colonization by the strain. However, as mentioned above, the effect of Cd^2+^ on the biocontrol efficiency of *B. subtilis* 1JN2 against *Ralstonia* was not significant. Therefore, we further explored the mechanism by which this strain can simultaneously control bacterial wilt and assist the host to alleviate cadmium stress.

The transcriptome sequencing results show that the expression levels of the EPS-encoding genes were significantly reduced, but that of γ-PGA genes increased after Cd^2+^ treatment. Further analysis revealed that KinC is the receptor of exogenous Cd^2+^ in *B. subtilis* 1JN2, which activates the main regulatory factor Spo0A through phosphorylation. This activation process is regulated by the critical protein Sda, which also controls sporulation or biofilm formation in bacterial cells. The decline in Spo0A levels inhibited the EPS synthesis through the SinI/SinR two-component regulatory system and increased AbrB levels. According to previous reports, metal ions generally positively regulate biofilm formation. For example, in *Salmonella enterica* and *Erwinia amylovora*, the regulator Zur specifically regulates the uptake of zinc ions and promotes the formation of bacterial biofilms (Ammendola et al., 2016; Kharadi and Sundin, 2020). For some pathogenic microorganisms, ion acquisition is critical for survival within the host. Therefore, specialized ion uptake regulators such as Fur and XibR can help these pathogens obtain enough metal ions and can regulate their adhesion and pathogenicity (Pandey et al., 2016; Teper et al., 2021).

Cadmium ion treatment significantly increased the expression of SigD, thus enhancing the expression of the flagella encoding and assembly genes of the strain. This further activated the DegS/DegU two-component regulatory system for γ-PGA production, enhancing the mobility of the strain and conversion of EPS to γ-PGA. Both EPS and γ-PGA have been reported to improve the colonization ability of *Bacillus* strains. However, in our results, we found that the bacterial population that colonized the tomato root surface was decreased. Accordingly, we speculate that the roles of γ-PGA and EPS in colonization are interchangeable in some *Bacillus* strains but not for strain 1JN2 on tomato root surfaces. As such, there may be other reasons why this strain can simultaneously control bacterial wilt and assist the host in alleviating cadmium stress.

The expression of surfactin synthesis-related genes was also significantly increased after Cd^2+^ treatment. Previous studies also reported that metal ions could induce surfactant secretion by *Bacillus* (López et al., 2009). As an important active protein product, surfactin plays a key role in alleviating heavy metal stress and controlling plant diseases (Almoneafy et al., 2014).According to our results, increased surfactin levels may improve the biocontrol ability of the strain by resisting metal ion stress and exerting inhibitory activity against *Ralstonia solanacearum*.

Generally, the main changes caused by Cd^2+^ treatment in *B. subtilis* 1JN2 biofilm were decreased EPS content, increased γ-PGA and surfactin levels, and enhanced cell mobility. Based on this, we deduce that the adaptive changes by *Bacillus* do not affect its disease control activity within a certain range of cadmium ion concentrations. Through surfactin secretion, *B. subtilis* can alleviate pathogen infections and heavy metal stress for the host plant. Exploring additional mechanisms involved in *B. subtilis* biofilm changes and screening of multifunctional biocontrol strains will be the focus of our future work.

## Data availability statement

The datasets presented in this study can be found in online repositories. The names of the repository/repositories and accession number(s) can be found in the article/Supplementary material.

## Author contributions

WY and YL designed the experiments. WY, HY, GD, ZL, and DZ performed the experiments. WY, CJ, DG, and DN analyzed the results and wrote the manuscript. All authors contributed to the article and approved the submitted version.

## Funding

This work was funded by the Natural Science Foundation for Higher Education Institutions of Jiangsu Province (21KJA210005), Natural Science Foundation of Jiangsu Province (BK20170467), and Jiangsu University Student Innovation Training Program (201910323013Z). These funding bodies had no role in the design of the study and collection, analysis, and interpretation of data and in writing the manuscript.

## Conflict of interest

The authors declare that the research was conducted in the absence of any commercial or financial relationships that could be construed as a potential conflict of interest.

## Publisher’s note

All claims expressed in this article are solely those of the authors and do not necessarily represent those of their affiliated organizations, or those of the publisher, the editors and the reviewers. Any product that may be evaluated in this article, or claim that may be made by its manufacturer, is not guaranteed or endorsed by the publisher.

## Footnotes

^1^ http://www.chgenomics.com

^2^ https://www.ncbi.nlm.nih.gov/search/all/?term=PRJNA646606

## Supplementary material

The Supplementary material for this article can befound online at: https://www.frontiersin.org/articles/10.3389/fmicb.2022.1002482/full#supplementary-material

SUPPLEMENTARY FIGURE S1 Colonization of *B. subtilis* 1JN2 on the root of tomato with **(C)** or without **(B)** Cd^2+^ treatment.

SUPPLEMENTARY FIGURE S2 PCA analysis of the differentially expressed genes of *B. subtilis* 1JN2 after Cd^2+^ treatment. Samples collected at 6h, 12h, 18h, and 24h were named as S2, S3, S4, S5 and the blank control that without Cd^2+^ was named as S1.
