# Supplementary figures and images for: Comparative transcriptomics reveal different genetic adaptations of biofilm formation in Bacillus subtilis isolate 1JN2 in response to Cd2+ treatment
Source: Front Microbiol. 2022 Oct 4;13:1002482. doi: 10.3389/fmicb.2022.1002482 (PMC9577173; doi:10.3389/fmicb.2022.1002482)

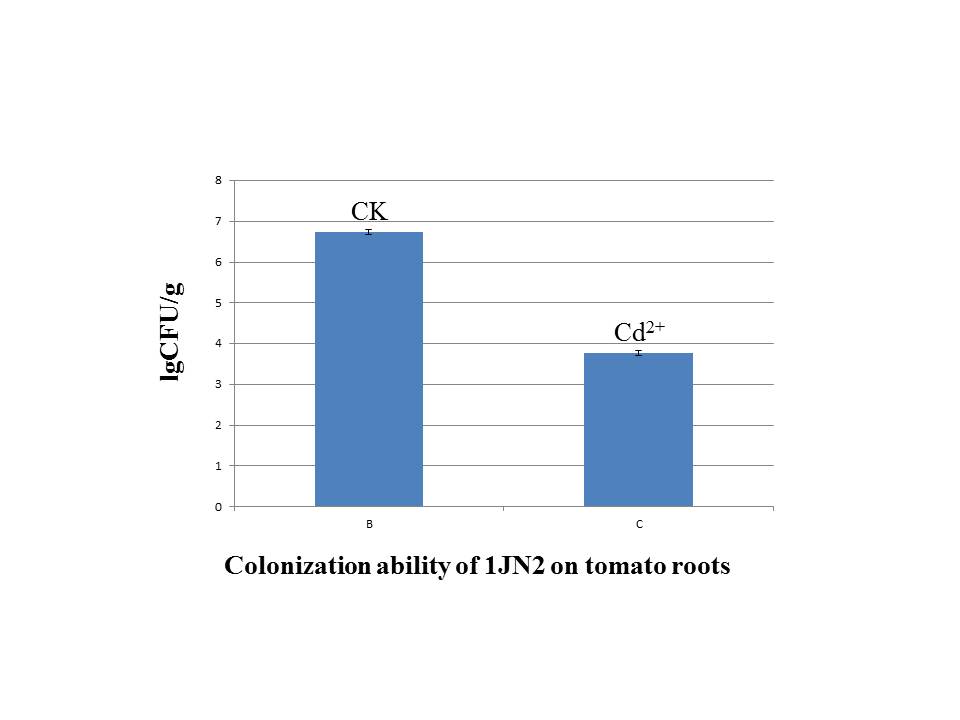

Supplement: SUPPLEMENTARY FIGURE S1 — Colonization of B. subtilis 1JN2 on the root of tomato with (C) or without (B) Cd2+ treatment. [file Image_1.jpg]

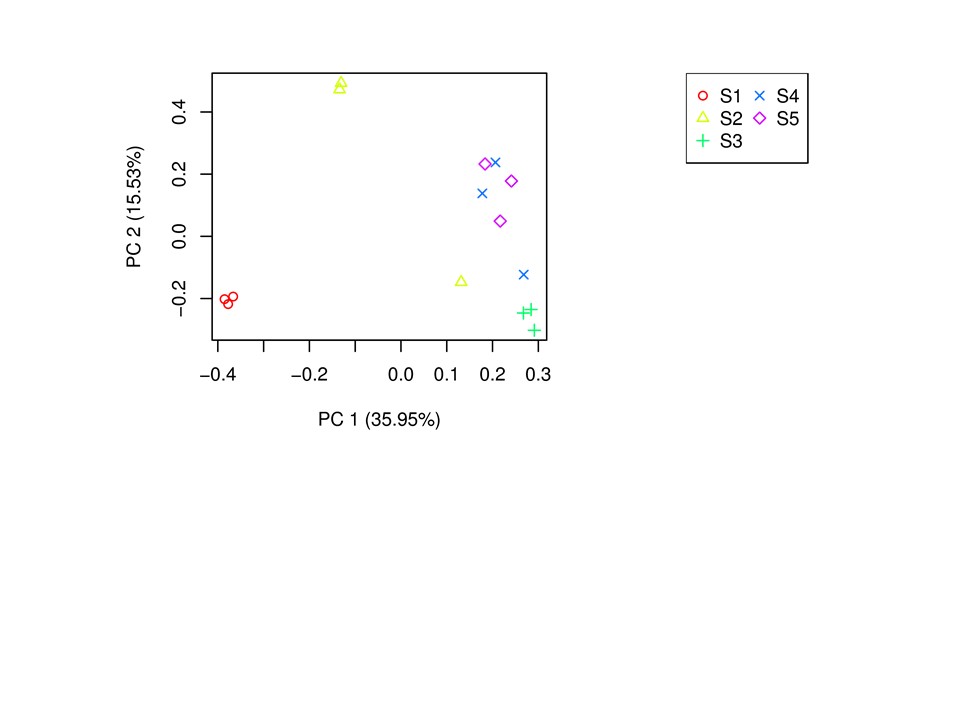

Supplement: SUPPLEMENTARY FIGURE S2 — PCA analysis of the differentially expressed genes of B. subtilis 1JN2 after Cd2+ treatment. Samples collected at 6h, 12h, 18h, and 24h were named as S2, S3, S4, S5 and the blank control that without Cd2+ was named as S1. [file Image_2.jpg]
