# Supplementary material for: Comparative transcriptomics reveal different genetic adaptations of biofilm formation in Bacillus subtilis isolate 1JN2 in response to Cd2+ treatment
Source: Front Microbiol. 2022 Oct 4;13:1002482. doi: 10.3389/fmicb.2022.1002482 (PMC9577173; doi:10.3389/fmicb.2022.1002482)
Supplement: Supplementary file 3 [file Table_1.docx]

Table 1 Impacts of Cd^2+^ on the biofilm colonies of *B. subtilis* 1JN2

| Cd（mM） | Diameter（mm） | Height（mm） |
| --- | --- | --- |
| 0 | 18.67±0.33a | 2.91±0.06a |
| 1 | 18.33±0.30a | 2.47±0.03b |
| 2 | 16.33±0.23b | 2.20±0.06c |
| 3 | 13.67±0.29c | 1.64±0.09d |
| 4 | 14.33±0.22c | 1.17±0.09e |
| 5 | 13.33±0.19c | 0.90±0.06f |
